# Hospital Consumer Assessment of Healthcare Providers and Systems survey response rates are significantly affected by patient characteristics and postoperative outcomes for patients undergoing primary total knee arthroplasty

**DOI:** 10.1371/journal.pone.0257555

**Published:** 2021-09-28

**Authors:** Michael R. Mercier, Anoop R. Galivanche, Wyatt B. David, Rohil Malpani, Neil Pathak, Ari S. Hilibrand, Lee E. Rubin, Jonathan N. Grauer

**Affiliations:** Department of Orthopaedics and Rehabilitation, Yale School of Medicine, New Haven, CT, United States of America; Ohio State University Wexner Medical Center Department of Surgery, UNITED STATES

## Abstract

**Introduction:**

The Hospital Consumer Assessment of Healthcare Providers and Systems (HCAHPS) survey measures patients’ satisfaction of their hospital experience. A minority of discharged patients return the survey. Underlying bias among who ultimately returns the survey (non-response bias) after total knee arthroplasty (TKA) may affect results of the survey. Thus, the objective of the current study is to assess the relationship between patient characteristics and postoperative outcomes on HCAHPS survey nonresponse.

**Methods:**

All adult patients at a single institution undergoing inpatient, elective, primary TKA between February 2013 and May 2020 were selected for analysis. Following discharge, all patients had been mailed the HCAHPS survey. The primary outcome analyzed in the current study is survey return. Patient characteristics, surgical variables, and 30-day postoperative outcomes were analyzed. Univariate and multivariate analyses were performed to identify factors independently associated with return of the HCAHPS survey.

**Results:**

Of 4,804 TKA patients identified, 1,498 (31.22%) returned HCAHPS surveys. On multivariate regression analyses controlling for patient factors, patients who did not return the survey were more likely to have a higher American Society of Anesthesia score (ASA score of 4 or higher, OR = 2.37; P<0.001), and be partially or totally dependent (OR = 2.37; P = 0.037). Similarly, patients who did not return the survey were more likely to have had a readmission (OR = 1.94; P<0.001), be discharged to a place other than home (OR = 1.52; P<0.001), or stay in the hospital for longer than 3 days (OR = 1.43; P = 0.004).

**Discussion:**

Following TKA, HCAHPS survey response rate was only 31.22% and completion of the survey was associated with several demographic and postoperative variables. These findings suggest that HCAHPS survey results capture a non-representative fraction of the true TKA patient population. This bias is necessary to consider when using HCAHPS survey results as a metric for quality of healthcare and federal reimbursement rates.

## Introduction

As administrative efforts continue to focus on making healthcare more patient-centered, it has become increasingly important to have valid tools for measuring patient perceptions of healthcare [1–3]. One notable recognition of this has been the introduction of the Hospital Consumer Assessment of Healthcare Providers and Systems (HCAHPS) survey.

The Centers for Medicare & Medicaid Services (CMS) developed the HCAHPS survey to standardize hospital assessment and create a means by which patients could easily compare care at different facilities [4]. As part of the growing movement to emphasize the patient experience, the HCAHPS survey quickly gained support and became tied to the Annual Payment Update (APU) for the Inpatient Prospective Payment System (IPPS) in 2008 [5]. This relationship meant that failure to collect and report HCAHPS scores could result in a decrease of up to 2% of a hospital’s APU. Further, under the Value-Based Purchasing (VBP) program, hospitals are financially rewarded for both achievement (as measured by a hospital’s HCAHPS score percentile across all hospitals) and improvement (changes in a hospital’s HCAHPS scores over time [6].

While marked efforts were made to develop a survey that accurately reflected quality of care, the correlation between HCAHPS survey results and patient outcomes is controversial, and may be influenced by patient demographics. For example, a study of HCAHPS survey results of total knee arthroplasty (TKA) patients at a single institution found that there was no significant correlation between overall hospital rating scores and a variety of validated patient reported outcome measures (PROM’s), including EuroQol-EQ-5D Instrument and Hip Disability and Osteoarthritis Outcome Score [7]. A total hip arthroplasty (THA) study found that patients who were male, African American, of lower socioeconomic status, and had shorter lengths of stay were more likely to report higher levels of inpatient satisfaction [8].

Perhaps a larger problem with the HCAHPS survey is the possibility of a non-response bias. This occurs when certain subgroups of a population have lower response rates to the survey, potentially skewing the results [9]. As CMS estimates that only 25% of eligible discharges will respond, there is a large potential for such a bias [10]. In fact, non-response biases have already been documented in a Press Ganey survey study in which demographic factors were shown to influence response rates [11].

TKA is among the highest volume orthopedic procedures performed annually. Currently, little is known regarding how HCAHPS survey response rates may be affected by patient characteristics and postoperative variables for patients undergoing TKA. Identification of factors contributing to a potential nonresponse bias are crucial in analyzing HCAHPS survey results, provide context for their interpretation, and guide ways in which survey response rates can be improved upon. Thus, the purpose of the current study is to examine the potential correlation of these variables with HCAHPS responses rates after TKA.

## Methods

### Patient inclusion/exclusion criteria

All patients who underwent, inpatient, elective TKA at a single institution and were sent the HCAHPS questionnaire from February 2013–May 2020 were selected for analysis. Patients were identified using the Current Procedural Terminology (CPT) code for primary total knee arthroplasty, 27447.

Patients less than 18 years of age, patients that died during their hospital stay, and patients undergoing revision or partial knee arthroplasty were excluded from the study. HCAHPS responses resulting from any patient readmission encounters were not separately assessed. Prior to the intiation of any data acquisition or analysis, this study was approved by our institution’s Institutional Review Board (IRB).

### Data elements

Patient demographic, preoperative, and 30-day postoperative outcome data is recorded systematically by our institution’s National Surgical Quality Improvement Program (NSQIP) team for all TKA patients. This team is composed of trained clinicians that track patients for 30 days postoperatively to characterize patient characteristics and procedures, as well as document the incidence of postoperative complications, readmissions, and additional surgeries [12].

Data elements abstracted included: patient age, sex, height and weight (used to calculate body mass index [BMI]), American Society of Anesthesia (ASA) score, functional health status, and race. ASA score data was utilized as a proxy for patient comorbidity burden, as is often done in orthopedics literature [13, 14]. The treating surgeon for each patient was also noted, to determine if there was an association between nonresponse bias and treating clinician.

Postoperative adverse events abstracted were categorized as major or minor adverse events. Major adverse events include: deep infection, sepsis and septic shock, ventilator use greater than 48 hours, unplanned intubation, acute renal failure, deep vein thrombosis, pulmonary embolism, cardiac arrest, myocardial infarction, and stroke. Minor adverse events include: superficial infection, wound dehiscence, pneumonia, urinary tract infection, and renal insufficiency. In addition to the aforementioned postoperative events, readmission within 30 days, discharge disposition (home or other), and prolonged hospital stay of greater than three days, were separately tabulated and analyzed.

All patients who have a total knee arthroplasty performed at our institution are mailed an HCAHPS survey within 2 days of discharge from our institution. Survey responses are then mailed back to our institution. All returned HCAHPS surveys were accounted for when calculating survey return rate, regardless of if the survey was fully completed by the patient.

### Statistical analysis

All aforementioned variables were compared between patients who returned the HCAHPS survey versus those who did not. Univariate analysis of categorical demographic and postoperative variables were compared using chi-squared or Fisher’s exact tests. Continuous demographic variables were compared using Student’s t-test. Pearson Correlation Coefficient was calculated to assess for potential linear association between surgeon case volume and survey response rate.

Lastly, binary logistic regressions were performed to assess for independent correlation of patient and postoperative variables on HCAHPS survey return. The first regression included the following patient demographic factors: age, sex, BMI, ASA class, preoperative functional status, and race. The second regression included the following postoperative factors: any, major, and minor adverse event occurrence, readmission, hospital discharge destination, and prolonged hospital stay. All covariates utilized in the first regression were included in the second regression.

Statistical significance was set at α = 0.05, and 95% confidence intervals [CI] were reported. Statistical analysis was performed with IBM SPSS Statistics, version 27 (IBM Corp., Armonk, N.Y., USA).

## Results

### Study population

In total, 4,804 TKA patients were identified. Of these, HCAHPS survey was returned by 1,498 (31.22%). Postoperatively, patients who did not return the survey varied by attending surgeon, with the highest response rate being 39% for surgeon 1 and 21% for surgeon 7 (p<0.001).

Notably, no statistically significant correlation was observed between surgeon volume and HCAHPS response rate (p = 0.093).

### Patient and postoperative outcome variables

In terms of patient factors, univariate chi-squared analysis revealed that those who did not return the survey were more likely to be younger (mean of 66.46 years compared with 68.35 years, p<0.001), have slightly higher BMI’s (mean of 32.80 compared with 31.48, p<0.001) have higher ASA class scores (46.01% of patients with an ASA score of 3 compared to 32.04%, p<0.001), be slightly less functionally independent (98.46% functionally independent compared with 99.53%, p = 0.002), and be non-white (74.68% white compared with 90.85% white, p<0.001). These findings are summarized in Table 1.

**Table 1:** Demographics of patients undergoing primary TKA organized by status of HCAHPS survey return.

Survey Status	Survey Not Returned	Survey Returned	Univariate P-value
N = 4,804 (100%)	N = 3,306 (68.82%)	N = 1,498 (31.22%)	
**Age: Mean [SD]**	**66.46 [10.06]**	**68.35 [8.59]**	**<0.001**
18–34	13 (.39%)	1 (0.07%)	
35–54	387 (11.71%)	93 (6.21%)	
55–74	2,217 (67.06%)	1,065 (71.09%)	
≥ 75	689 (20.84%)	339 (22.63%)	
**Sex**			0.859
Male	1,205 (36.45%)	550 (36.72%)	
Female	2,101 (63.55%)	948 (63.28%)	
**BMI: Mean [SD]**	**32.80 [6.94]**	**31.48 [6.16]**	**<0.001**
< 25	369 (11.16%)	195 (13.02%)	
25–30	906 (27.40%)	466 (31.11%)	
30–35	901 (27.25%)	449 (29.97%)	
> 35	1,122 (33.94%)	382 (25.50%)	
**ASA**			**<0.001**
1	96 (2.90%)	74 (4.94%)	
2	1,640 (49.61%)	936 (62.48%)	
3	1,521 (46.01%)	480 (32.04%)	
4+	49 (1.48%)	7 (0.47%)	
**Functional Status (prior to surgery):**			**0.002**
Independent	3,255 (98.46%)	1,491 (99.53%)	
Partially/Totally dependent	51 (1.54%)	7 (0.47%)	
**Race**			**<0.001**
White	2,469 (74.68%)	1,361 (90.85%)	
Black/African American	582 (17.60%)	93 (6.21%)	
Asian	29 (0.88%)	11 (0.73%)	

**Bolding** indicates statistical significance at p<0.05.

In terms of postoperative variables, univariate chi-squared analysis revealed that those who did not return the survey were more likely to experience: a minor adverse event (2.51% compared with 1.47%, p = 0.022), pneumonia (0.91% compared with 0.13%, p = 0.002), readmission (6.26% compared with 3.00%, p<0.001), and be discharged to a place other than home (40.29% compared with 27.70%, p<0.001). These findings are summarized in Table 2.

**Table 2:** Adverse event outcomes following primary TKA organized by status of HCAHPS survey return.

Survey Status	Survey Not Returned	Survey Returned	Univariate P-value
N = 4,804 (100%)	N = 3,306 (68.82%)	N = 1,498 (31.22%)	
All Adverse Events	170 (5.14%)	64 (4.27%)	0.195
**Major Adverse Events**	99 (2.99%)	47 (3.14%)	0.789
Deep Infection	3 (0.09%)	1 (0.07%)	0.789
Sepsis/Septic shock	12 (0.36%)	4 (0.27%)	0.593
Ventilator >48 hours	3 (0.09%)	0 (0.00%)	0.244
Unplanned Intubation	4 (0.12%)	2 (0.13%)	0.909
Acute Renal Failure	1 (0.03%)	0 (0.00%)	0.501
Deep Vein Thrombosis	44 (1.33%)	20 (1.34%)	0.991
Pulmonary Embolism	43 (1.30%)	25 (1.67%)	0.317
Cardiac Arrest	0 (0.00%)	1 (0.07%)	0.137
MI	7 (0.21%)	1 (0.07%)	0.254
Stroke	2 (0.06%)	2 (0.13%)	0.416
**Minor Adverse Events**	**83 (2.51%)**	**22 (1.47%)**	**0.022**
Superficial Infection	24 (0.73%)	6 (0.40%)	0.185
Wound Disruption	1 (0.03%)	1 (0.07%)	0.566
**Pneumonia**	**30 (0.91%)**	**2 (0.13%)**	**0.002**
UTI	29 (0.88%)	13 (0.87%)	0.974
Progressive Renal Insufficiency	5 (0.15%)	0 (0.00%)	0.132
**Readmissions**	**207 (6.26%)**	**45 (3.00%)**	**<0.001**
**Discharge Disposition**			**<0.001**
Home	1,974 (59.71%)	1,083 (72.30%)	
Other	1,332 (40.29%)	415 (27.70%)	
**Long hospital length of stay (> 3 days)**	24 (.73%)	6 (.40%)	0.185

### Multivariate analyses

**Fig 1:**
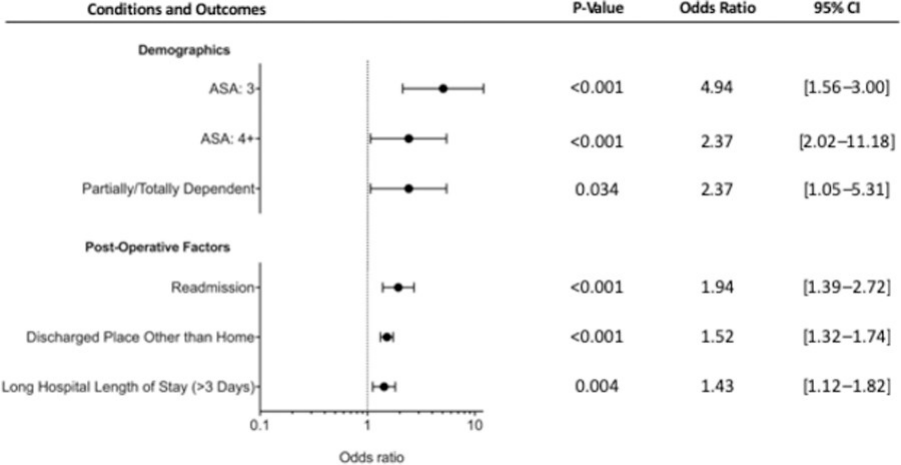
Binary logistic regression: Factors independently associated with HCAHPS survey nonresponse. Forest plot depicting the significant variables from the multivariate regression on both demographic and post-operative factors highlighting HCAHPS non-responder bias following total knee arthroplasty.

Based on multivariate analysis controlling for patient factors, there was no association between age, sex, BMI, or race and non-response (p>0.05 for all). With regards to ASA score, using the patient cohort with ASA classifications of 1 as the referent, there were increased odds of non-response in patients with an ASA of 3 (OR = 4.94; 95% CI, 2.09–11.68; p<0.001) and 4+ (OR = 2.37; 95% CI, 1.05–5.31; p = 0.037). Patients with non-independent pre-operative functional statuses had increased odds of non-response compared to patients who had independent pre-operative functional statuses (OR = 2.37; 95% CI, 1.05–5.31; p = 0.037). These findings are summarized in Table 3, and statistically significant findings are shown in Fig 1.

**Table 3:** Demographic factors associated with not returning the HCAHPS survey in patients undergoing primary TKA.

N = 4,804 (100%)	Likelihood of Not Returning Survey		
				OR	95% CI	P-value
**Age**			
18–34	1.00	–	–
35–54	0.36	[0.05–2.81]	0.327
55–74	0.20	[0.03–1.56]	0.124
75+	0.20	[0.03–1.59]	0.129
**Sex**			
Female	1.00	–	**–**
Male	1.06	[0.93–1.21]	0.375
**BMI**			
<25	1.00	–	**–**
25–30	0.98	[0.79–1.21]	0.850
30–35	0.89	[0.72–1.11]	0.307
>35	1.05	[0.84–1.32]	0.672
**ASA**			
1	1.00	–	**–**
2	1.29	[0.93–1.77]	0.125
** 3**	**4.94**	**[2.09–11.68]**	**<0.001**
** 4+**	**2.37**	**[1.05–5.31]**	**<0.001**
**Functional Status (Prior to Surgery)**			
Independent	1.00	–	–
**Partially/Totally Dependent**	**2.37**	**[1.05–5.31]**	**0.037**
**Race**			
White	1.00	–	**–**
Black or African American	1.53	[0.76–3.09]	0.237

**Factors in Model:** All Factors in Table 1.

Based on multivariate analysis controlling for patient and 30-day postoperative outcome variables, there were increased odds of non-response in patients who experienced hospital readmission (OR = 1.94; 95% CI, 1.39–2.72; p<0.001), those who were discharged to a place other than home (OR = 1.52; 95% CI, 1.32–1.74; p<0.001), and those who experienced a prolonged hospital stay (OR = 1.43; 95% CI, 1.12–1.82; p<0.001). There was no statistically significant difference in odds of non-response in patients who experienced any adverse event, a major adverse event, or a minor adverse event (p>0.05 for all). These findings summarized in Table 4, and statistically significant findings are shown in Fig 1.

**Table 4:** Post-operative factors independently associated with not returning the HCAHPS survey in patient undergoing primary total knee replacement.

N = 4,804 (100%)	Likelihood of Not Returning Survey		
				OR	95% CI	P-value
Any Adverse Event	1.16	[0.86–1.57]	0.340
Major Adverse Event	0.94	[0.65–1.35]	0.741
Minor Adverse Event	1.54	[0.95–2.50]	0.082
**Readmission**	**1.94**	**[1.39–2.72]**	**<0.001**
**Discharged Place Other Than Home**	**1.52**	**[1.32–1.74]**	**<0.001**
**Long Hospital Length of Stay (> 3 Days)**	**1.43**	**[1.12–1.82]**	**0.004**

**Factors in Model:** All Factors in Tables 1 and 2.

## Discussion

HCAHPS scores were implemented to assess patient satisfaction following hospitalization. Nonetheless, results of such surveys may be subject to non-response bias. As a high-volume elective procedure, HCAHPS responses rates were assessed for those undergoing TKA in the current study. Here, we demonstrate that higher ASA score, functional dependence, hospital readmission, discharge destination, and prolonged hospital stay were independently associated with HCAHPS survey nonresponse.

An HCAHPS satisfaction survey return rate of 31.22% observed in the current study after TKA, which was slightly above the overall national return rate of 25% [10]. Based on univariate analyses, it was found that patients who did not return the survey were slightly older, had higher BMI and ASA scores, and were more likely to be functionally dependent prior to surgery. These patients have traditionally had poorer outcomes following surgery suggesting that missing their input could bias survey outcomes [15–17]. Additionally, survey non-responders were found to be more non-white. This finding is concordant with prior literature, as minorities have been noted to both return the HCAHPS surveys at a lower rate, particularly notable as they typically return the survey with lower scores [18, 19].

To further investigate the reason for not completing the satisfaction survey, variability based on attending physician was investigated. Significant variation was seen based on the surgeon performing the operation. However, HCAHPS survey response rate did not correlate with surgeon case volume in the currently performed study. This finding is concordant with prior research which showed no apparent correlation between survey response rates and volume of total hip arthroplasty performed by the treating surgeon [20]. The role of physicians and staff in encouraging HCAHPS survey completion should be studied further to help identify factors leading to high return rates, and ways they can be modified to improve survey yield.

Being classified as a 3 or 4 on the ASA scale prior to surgery was associated with lower rates of HCAHPS survey return on univariate analysis. Similarly, patients who had partial or total functional status prior to surgery also demonstrated a higher incidence of survey nonresponse. These findings were recapitulated on multivariate analysis after controlling for patient demographic factors, which strengthen the aforementioned pre-existing associations observed on univariate analysis. This is yet another example of non-response bias leading to skewed HCAHPS satisfaction data and exemplifies the necessity of improving response rates for all patient cohorts.

Furthermore, the current study demonstrated that several postoperative events, such as readmission, discharge to a place other than home, and long hospital length of stay, were shown to correlate with nonresponse bias. It is plausible that patients who undergo more strenuous postoperative courses are less compelled to complete and return extra post-discharge paperwork such as the HCAHPS survey.

Additionally, patients discharged to a non-home destination may not have timely access to the survey in the event the survey was mailed home. While potentially related to the mechanics of receiving and returning the survey at a non-home site, the responses of these patients is important and should methods to address this should be considered. In the context of this finding, current HCAHPS data may be disproportionately representing patients with less arduous postoperative courses, who may have a different perception of their healthcare quality than nonresponders.

Prior studies have examined nonresponse bias in a total hip arthroplasty (THA) population [20]. Several studies have highlighted crucial differences between THA and TKA patients in terms of demographics and clinical outcomes, which could conceivably influence survey responses [21–23]. Unlike in the prior THA cohort, there were no statistically significant associations between demographic factors such as older age and race and response rates in the present TKA population. The current study also did not find an association between the occurrence of postoperative adverse events and survey nonresponse.

The current TKA and prior THA findings also shared several similarities. Survey response rates were similar between the present TKA and prior THA cohorts. Furthermore, discharge disposition, readmission, and long hospital length of stay were recurring predictors of survey nonresponse in both studies. These shared findings highlight the importance of discharge planning and postoperative optimization on survey response rates following surgery in total joint arthroplasty patients [20].

The current study highlights the need to increase response HCAHPS response rate to capture a more representative patient sample. Nationally, HCAHPS surveys are currently distributed via four different survey modes, including mail, mail, telephone, mail with telephone follow-up, or active interactive voice recognition [24]. At our institution, all surveys during the study period were sent to patients by mail. Hospitals may benefit by offering more delivery modalities, such as a digital mode of delivery. Future research should aim to identify patient-specific barriers to survey response. Notable barriers that should be considered include comprehension issues among non-English speaking or illiterate patients, a discordance between the survey mailing address and the patient’s discharge destination, and inability to mail in the survey due to functional limitations. Once specific reasons for survey nonresponse are identified, specific strategies can be implemented to improve response rate.

Notably, the current study has limitations. The data originates from one institution and may affect generalizability. Furthermore, adverse outcomes only followed for thirty days following operation, so the effect of later events could have been missed. Finally, the retrospective nature of the current study lacks the robustness of a prospective study.

Conversely, the current study has numerous strengths. To the authors’ knowledge, the current study represents the largest sample to date examining HCAHPS responses for a single-institution TKA cohort. Demographic and post-operative characteristics were analyzed using multivariate logistic regressions to independently determine the influence of multiple factors on the rate of survey completion. Additionally, this study specifically targeted attention to the response rate to HCAHPS survey as opposed to the grading of these responses.

## Conclusions

Overall, for TKA, this study confirmed that demographic factors and post-operative outcomes were significantly associated with HCAHPS satisfaction survey response rate. These conclusions are important factors to remain cognizant of when interpreting HCAHPS survey data, and the use of it when drawing conclusions on patient satisfaction from a potentially biased subgroup of TKA patients.

## Supporting information

S1 Dataset (XLSX)
